# Supplementary material for: Modeling glioblastoma heterogeneity as a dynamic network of cell states
Source: Mol Syst Biol. 2021 Sep 16;17(9):e10105. doi: 10.15252/msb.202010105 (PMC8444284; doi:10.15252/msb.202010105)
Supplement: Supplementary file 6 — Source Data for Figure 5 [file MSB-17-e10105-s004.zip › Figure5A_sourcedata/GSEA_3071/nosigdb_stateII.GseaPreranked.1622795151171/gsea_report_for_na_neg_1622795151171.html]

Report for na\_neg 1622795151171 [GSEA]

| GS  follow link to MSigDB | GS DETAILS | SIZE | ES | NES | NOM p-val | FDR q-val | FWER p-val | RANK AT MAX | LEADING EDGE || 1 | LARSSON\_STATE\_6 | Details ... | 73 | -0.69 | -3.66 | 0.000 | 0.000 | 0.000 | 1004 | tags=84%, list=24%, signal=109% |
| 2 | RICHARDS\_NOWAKOWSKI\_SCIENCE\_2017\_ASTROCYTE\_UPREG | Details ... | 290 | -0.53 | -3.52 | 0.000 | 0.000 | 0.000 | 725 | tags=46%, list=18%, signal=52% |
| 3 | RICHARDS\_ZHONG\_NATURE\_2018\_OPC\_UPREG | Details ... | 49 | -0.73 | -3.48 | 0.000 | 0.000 | 0.000 | 582 | tags=65%, list=14%, signal=75% |
| 4 | RICHARDS\_NOWAKOWSKI\_SCIENCE\_2017\_OPC\_UPREG | Details ... | 196 | -0.55 | -3.44 | 0.000 | 0.000 | 0.000 | 592 | tags=44%, list=14%, signal=49% |
| 5 | RICHARDS\_NOWAKOWSKI\_SCIENCE\_2017\_NIN1\_UPREG | Details ... | 94 | -0.62 | -3.36 | 0.000 | 0.000 | 0.000 | 853 | tags=66%, list=21%, signal=81% |
| 6 | RICHARDS\_NOWAKOWSKI\_SCIENCE\_2017\_IPC\_NEN1\_UPREG | Details ... | 141 | -0.56 | -3.30 | 0.000 | 0.000 | 0.000 | 959 | tags=61%, list=23%, signal=77% |
| 7 | RICHARDS\_NEFTEL\_CELL\_2019\_NPC1 | Details ... | 31 | -0.76 | -3.20 | 0.000 | 0.000 | 0.000 | 549 | tags=71%, list=13%, signal=81% |
| 8 | SUVA\_NPC1\_PMID\_31327527 | Details ... | 31 | -0.76 | -3.15 | 0.000 | 0.000 | 0.000 | 549 | tags=71%, list=13%, signal=81% |
| 9 | RICHARDS\_ZHONG\_NATURE\_2018\_ASTROCYTES\_UPREG | Details ... | 146 | -0.53 | -3.11 | 0.000 | 0.000 | 0.000 | 587 | tags=41%, list=14%, signal=46% |
| 10 | RICHARDS\_VERHAAK\_CANCERCELL\_2010\_PRONEURAL | Details ... | 69 | -0.61 | -3.10 | 0.000 | 0.000 | 0.000 | 541 | tags=51%, list=13%, signal=57% |
| 11 | RICHARDS\_NOWAKOWSKI\_SCIENCE\_2017\_VRG\_UPREG | Details ... | 227 | -0.49 | -3.10 | 0.000 | 0.000 | 0.000 | 725 | tags=44%, list=18%, signal=51% |
| 12 | NB\_KI\_CLUSTER2 | Details ... | 148 | -0.51 | -3.09 | 0.000 | 0.000 | 0.000 | 1115 | tags=64%, list=27%, signal=85% |
| 13 | RICHARDS\_NOWAKOWSKI\_SCIENCE\_2017\_NIN3\_UPREG | Details ... | 81 | -0.59 | -3.08 | 0.000 | 0.000 | 0.000 | 508 | tags=47%, list=12%, signal=52% |
| 14 | RICHARDS\_NOWAKOWSKI\_SCIENCE\_2017\_IN\_CTX\_MGE1\_UPREG | Details ... | 153 | -0.50 | -3.02 | 0.000 | 0.000 | 0.000 | 696 | tags=44%, list=17%, signal=52% |
| 15 | RICHARDS\_NOWAKOWSKI\_SCIENCE\_2017\_NEN\_EARLY2\_UPREG | Details ... | 221 | -0.47 | -3.00 | 0.000 | 0.000 | 0.000 | 1015 | tags=52%, list=25%, signal=66% |
| 16 | DOROSZKO\_U3054MG\_CLUSTER\_0 | Details ... | 43 | -0.65 | -2.95 | 0.000 | 0.000 | 0.000 | 587 | tags=56%, list=14%, signal=64% |
| 17 | RICHARDS\_NOWAKOWSKI\_SCIENCE\_2017\_IN\_CTX\_CGE2\_UPREG | Details ... | 116 | -0.52 | -2.94 | 0.000 | 0.000 | 0.000 | 678 | tags=46%, list=17%, signal=53% |
| 18 | RICHARDS\_NOWAKOWSKI\_SCIENCE\_2017\_IN\_CTX\_MGE2\_UPREG | Details ... | 118 | -0.51 | -2.93 | 0.000 | 0.000 | 0.000 | 877 | tags=55%, list=21%, signal=68% |
| 19 | RICHARDS\_NOWAKOWSKI\_SCIENCE\_2017\_EN\_PFC3\_UPREG | Details ... | 126 | -0.51 | -2.91 | 0.000 | 0.000 | 0.000 | 563 | tags=40%, list=14%, signal=45% |
| 20 | RICHARDS\_NEFTEL\_CELL\_2019\_NPC2 | Details ... | 28 | -0.72 | -2.89 | 0.000 | 0.000 | 0.000 | 546 | tags=68%, list=13%, signal=78% |
| 21 | LARSSON\_STATE\_2 |  | 53 | -0.60 | -2.88 | 0.000 | 0.000 | 0.000 | 348 | tags=40%, list=8%, signal=43% |
| 22 | SUVA\_NPC2\_PMID\_31327527 |  | 28 | -0.72 | -2.87 | 0.000 | 0.000 | 0.000 | 546 | tags=68%, list=13%, signal=78% |
| 23 | GAROFANO\_IAVARONE\_NEU\_SUBTYPE |  | 94 | -0.53 | -2.86 | 0.000 | 0.000 | 0.000 | 1173 | tags=66%, list=29%, signal=90% |
| 24 | OLSEN\_SCPLIKE |  | 46 | -0.62 | -2.85 | 0.000 | 0.000 | 0.000 | 931 | tags=74%, list=23%, signal=95% |
| 25 | RICHARDS\_NOWAKOWSKI\_SCIENCE\_2017\_IPC\_NEN3\_UPREG |  | 112 | -0.50 | -2.84 | 0.000 | 0.000 | 0.000 | 900 | tags=53%, list=22%, signal=66% |
| 26 | RICHARDS\_NEFTEL\_CELL\_2019\_OPC |  | 22 | -0.74 | -2.83 | 0.000 | 0.000 | 0.000 | 587 | tags=68%, list=14%, signal=79% |
| 27 | NB\_KI\_CLUSTER4 |  | 65 | -0.56 | -2.82 | 0.000 | 0.000 | 0.000 | 697 | tags=49%, list=17%, signal=58% |
| 28 | RICHARDS\_NOWAKOWSKI\_SCIENCE\_2017\_IN\_CTX\_CGE1\_UPREG |  | 128 | -0.48 | -2.81 | 0.000 | 0.000 | 0.000 | 743 | tags=45%, list=18%, signal=54% |
| 29 | SUVA\_AC\_PMID\_31327527 |  | 22 | -0.73 | -2.79 | 0.000 | 0.000 | 0.000 | 503 | tags=64%, list=12%, signal=72% |
| 30 | RICHARDS\_NOWAKOWSKI\_SCIENCE\_2017\_EN\_PFC2\_UPREG |  | 387 | -0.40 | -2.79 | 0.000 | 0.000 | 0.000 | 886 | tags=41%, list=22%, signal=47% |
| 31 | RICHARDS\_NOWAKOWSKI\_SCIENCE\_2017\_NIN5\_UPREG |  | 136 | -0.47 | -2.78 | 0.000 | 0.000 | 0.000 | 1104 | tags=57%, list=27%, signal=75% |
| 32 | DOROSZKO\_U3180MG\_CLUSTER\_2 |  | 231 | -0.43 | -2.77 | 0.000 | 0.000 | 0.000 | 1122 | tags=52%, list=27%, signal=67% |
| 33 | RICHARDS\_NEFTEL\_CELL\_2019\_AC |  | 22 | -0.73 | -2.77 | 0.000 | 0.000 | 0.000 | 503 | tags=64%, list=12%, signal=72% |
| 34 | DOROSZKO\_U3013MG\_CLUSTER\_0 |  | 26 | -0.68 | -2.74 | 0.000 | 0.000 | 0.000 | 257 | tags=46%, list=6%, signal=49% |
| 35 | SUVA\_OPC\_PMID\_31327527 |  | 22 | -0.74 | -2.74 | 0.000 | 0.000 | 0.000 | 587 | tags=68%, list=14%, signal=79% |
| 36 | DOROSZKO\_PERIVASCULAR\_INVASION |  | 28 | -0.66 | -2.72 | 0.000 | 0.000 | 0.000 | 438 | tags=50%, list=11%, signal=56% |
| 37 | ALLEN\_112\_ASTROL1FGFR3MT1G |  | 15 | -0.78 | -2.68 | 0.000 | 0.000 | 0.000 | 503 | tags=73%, list=12%, signal=83% |
| 38 | RICHARDS\_NOWAKOWSKI\_SCIENCE\_2017\_IPC\_NEN1\_UPREG\_1 |  | 73 | -0.50 | -2.67 | 0.000 | 0.000 | 0.000 | 476 | tags=38%, list=12%, signal=43% |
| 39 | RICHARDS\_NOWAKOWSKI\_SCIENCE\_2017\_EN\_PFC1\_UPREG |  | 393 | -0.38 | -2.66 | 0.000 | 0.000 | 0.000 | 1313 | tags=54%, list=32%, signal=71% |
| 40 | POLLEN\_OUTERRADIALGLIA\_PMID\_26406371 |  | 33 | -0.62 | -2.65 | 0.000 | 0.000 | 0.000 | 634 | tags=55%, list=15%, signal=64% |
| 41 | OUTERRADIALGLIA |  | 33 | -0.62 | -2.62 | 0.000 | 0.000 | 0.000 | 634 | tags=55%, list=15%, signal=64% |
| 42 | TISIG\_PMID\_32649888 |  | 26 | -0.67 | -2.62 | 0.000 | 0.000 | 0.000 | 442 | tags=50%, list=11%, signal=56% |
| 43 | ALLEN\_113\_ASTROL1FGFR3FOS |  | 15 | -0.76 | -2.60 | 0.000 | 0.000 | 0.000 | 503 | tags=73%, list=12%, signal=83% |
| 44 | RICHARDS\_NOWAKOWSKI\_SCIENCE\_2017\_EN\_V1\_1\_UPREG |  | 377 | -0.37 | -2.60 | 0.000 | 0.000 | 0.000 | 883 | tags=38%, list=22%, signal=44% |
| 45 | RICHARDS\_NOWAKOWSKI\_SCIENCE\_2017\_EN\_V1\_2\_UPREG |  | 319 | -0.38 | -2.59 | 0.000 | 0.000 | 0.000 | 1085 | tags=46%, list=26%, signal=58% |
| 46 | RICHARDS\_NOWAKOWSKI\_SCIENCE\_2017\_NIN4\_UPREG |  | 79 | -0.49 | -2.55 | 0.000 | 0.000 | 0.000 | 821 | tags=47%, list=20%, signal=57% |
| 47 | RICHARDS\_NOWAKOWSKI\_SCIENCE\_2017\_NEN\_LATE\_UPREG |  | 162 | -0.41 | -2.51 | 0.000 | 0.000 | 0.000 | 1165 | tags=51%, list=28%, signal=68% |
| 48 | OLSEN\_ADRENERGIC |  | 45 | -0.54 | -2.49 | 0.000 | 0.000 | 0.000 | 634 | tags=51%, list=15%, signal=60% |
| 49 | DOROSZKO\_U3013MG\_CLUSTER\_3 |  | 63 | -0.49 | -2.47 | 0.000 | 0.000 | 0.000 | 533 | tags=38%, list=13%, signal=43% |
| 50 | SINGH\_SOX2\_UPREGULATED\_TRANSCRIPTS\_PMID\_28122245 |  | 213 | -0.39 | -2.46 | 0.000 | 0.000 | 0.000 | 511 | tags=31%, list=12%, signal=34% |
| 51 | RICHARDS\_NOWAKOWSKI\_SCIENCE\_2017\_NIN2\_UPREG |  | 225 | -0.38 | -2.43 | 0.000 | 0.000 | 0.000 | 1642 | tags=70%, list=40%, signal=110% |
| 52 | TIISIG\_PMID\_32649888 |  | 17 | -0.69 | -2.43 | 0.000 | 0.000 | 0.000 | 314 | tags=47%, list=8%, signal=51% |
| 53 | RICHARDS\_NOWAKOWSKI\_SCIENCE\_2017\_MGE\_RG1\_UPREG |  | 161 | -0.40 | -2.42 | 0.000 | 0.000 | 0.000 | 561 | tags=30%, list=14%, signal=34% |
| 54 | RICHARDS\_NOWAKOWSKI\_SCIENCE\_2017\_EN\_V1\_3\_UPREG |  | 347 | -0.36 | -2.41 | 0.000 | 0.000 | 0.000 | 817 | tags=36%, list=20%, signal=42% |
| 55 | LARSSON\_STATE\_3 |  | 42 | -0.53 | -2.40 | 0.000 | 0.000 | 0.000 | 370 | tags=38%, list=9%, signal=41% |
| 56 | GAROFANO\_IAVARONE\_MTC\_SUBTYPE |  | 82 | -0.43 | -2.33 | 0.000 | 0.000 | 0.000 | 981 | tags=50%, list=24%, signal=64% |
| 57 | RICHARDS\_NOWAKOWSKI\_SCIENCE\_2017\_NEN\_EARLY1\_UPREG |  | 306 | -0.34 | -2.28 | 0.000 | 0.000 | 0.001 | 1142 | tags=44%, list=28%, signal=57% |
| 58 | GANDAL\_ALCOHOLADDICTION\_DN\_PMID29439242 |  | 31 | -0.54 | -2.26 | 0.000 | 0.000 | 0.002 | 466 | tags=35%, list=11%, signal=40% |
| 59 | RICHARDS\_NOWAKOWSKI\_SCIENCE\_2017\_IN\_STR\_UPREG |  | 87 | -0.42 | -2.25 | 0.000 | 0.000 | 0.002 | 1180 | tags=53%, list=29%, signal=73% |
| 60 | RICHARDS\_NOWAKOWSKI\_SCIENCE\_2017\_MGE\_IPC3\_UPREG |  | 79 | -0.42 | -2.22 | 0.000 | 0.000 | 0.003 | 765 | tags=46%, list=19%, signal=55% |
| 61 | FELDMAN\_2020\_M-EARLYG1 |  | 74 | -0.42 | -2.20 | 0.000 | 0.000 | 0.005 | 329 | tags=28%, list=8%, signal=30% |
| 62 | DOROSZKO\_U3054MG\_CLUSTER\_5 |  | 55 | -0.45 | -2.19 | 0.000 | 0.000 | 0.005 | 627 | tags=38%, list=15%, signal=44% |
| 63 | DOROSZKO\_U3054MG\_CLUSTER\_10 |  | 110 | -0.39 | -2.19 | 0.000 | 0.000 | 0.007 | 440 | tags=24%, list=11%, signal=26% |
| 64 | VANGRONINGEN\_ADRN |  | 157 | -0.36 | -2.17 | 0.000 | 0.000 | 0.012 | 759 | tags=41%, list=19%, signal=49% |
| 65 | ALMSTEDT\_ALK |  | 42 | -0.47 | -2.15 | 0.000 | 0.000 | 0.017 | 765 | tags=50%, list=19%, signal=61% |
| 66 | IVY\_IT |  | 28 | -0.52 | -2.13 | 0.000 | 0.000 | 0.023 | 664 | tags=43%, list=16%, signal=51% |
| 67 | DOROSZKO\_U3180MG\_CLUSTER\_5 |  | 18 | -0.58 | -2.04 | 0.004 | 0.001 | 0.059 | 479 | tags=39%, list=12%, signal=44% |
| 68 | GANDAL\_BIPOLAR\_DN\_PMID29439242 |  | 15 | -0.61 | -2.01 | 0.002 | 0.002 | 0.083 | 330 | tags=53%, list=8%, signal=58% |
| 69 | DOROSZKO\_U3054MG\_CLUSTER\_3 |  | 102 | -0.36 | -2.00 | 0.000 | 0.002 | 0.094 | 586 | tags=31%, list=14%, signal=36% |
| 70 | KRONA\_ROSEN\_WHITE\_MATTER\_INVASION |  | 117 | -0.34 | -1.96 | 0.000 | 0.002 | 0.132 | 623 | tags=31%, list=15%, signal=35% |
| 71 | RICHARDS\_CAHOY\_JNEUROSCI\_2009\_NEURON |  | 411 | -0.28 | -1.95 | 0.000 | 0.003 | 0.143 | 934 | tags=34%, list=23%, signal=39% |
| 72 | RICHARDS\_CAHOY\_JNEUROSCI\_2011\_ASTRO\_MATURE |  | 245 | -0.30 | -1.94 | 0.000 | 0.003 | 0.151 | 1127 | tags=41%, list=27%, signal=53% |
| 73 | DOROSZKO\_U3054MG\_CLUSTER\_8 |  | 271 | -0.29 | -1.93 | 0.000 | 0.003 | 0.158 | 1484 | tags=54%, list=36%, signal=78% |
| 74 | NB\_KI\_CLUSTER17 |  | 76 | -0.37 | -1.92 | 0.000 | 0.003 | 0.168 | 946 | tags=45%, list=23%, signal=57% |
| 75 | WENG\_STEMNESS\_PMID\_30982771 |  | 22 | -0.49 | -1.91 | 0.006 | 0.003 | 0.177 | 476 | tags=41%, list=12%, signal=46% |
| 76 | NB\_KI\_CLUSTER23 |  | 130 | -0.32 | -1.90 | 0.000 | 0.003 | 0.192 | 948 | tags=40%, list=23%, signal=50% |
| 77 | ALMSTEDT\_VGR\_DIFF |  | 383 | -0.28 | -1.90 | 0.000 | 0.003 | 0.192 | 739 | tags=30%, list=18%, signal=33% |
| 78 | RICHARDS\_VERHAAK\_CANCERCELL\_2010\_CLASSICAL |  | 61 | -0.38 | -1.89 | 0.000 | 0.004 | 0.204 | 1031 | tags=48%, list=25%, signal=63% |
| 79 | IVY\_LE |  | 25 | -0.44 | -1.77 | 0.015 | 0.009 | 0.447 | 879 | tags=48%, list=21%, signal=61% |
| 80 | ALMSTEDT\_RISK |  | 21 | -0.48 | -1.76 | 0.028 | 0.009 | 0.459 | 125 | tags=29%, list=3%, signal=29% |
| 81 | TF\_CENSUS\_TAIPALE |  | 378 | -0.25 | -1.74 | 0.000 | 0.011 | 0.528 | 619 | tags=23%, list=15%, signal=25% |
| 82 | RICHARDS\_CAHOY\_JNEUROSCI\_2014\_MOG\_POS |  | 305 | -0.25 | -1.68 | 0.000 | 0.017 | 0.689 | 962 | tags=32%, list=23%, signal=39% |
| 83 | DOROSZKO\_U3013MG\_CLUSTER\_1 |  | 25 | -0.42 | -1.64 | 0.030 | 0.022 | 0.784 | 1151 | tags=60%, list=28%, signal=83% |
| 84 | LARSSON\_STATE\_5 |  | 44 | -0.35 | -1.63 | 0.027 | 0.023 | 0.809 | 343 | tags=20%, list=8%, signal=22% |
| 85 | KLING\_GBM\_PHENO\_PMID\_27667176 |  | 89 | -0.30 | -1.60 | 0.023 | 0.028 | 0.875 | 367 | tags=19%, list=9%, signal=21% |
| 86 | RICHARDS\_CAHOY\_JNEUROSCI\_2013\_OL\_MYEL |  | 468 | -0.23 | -1.59 | 0.001 | 0.029 | 0.890 | 1342 | tags=44%, list=33%, signal=58% |
| 87 | GANDAL\_SCHIZOPHRENIA\_UP\_PMID29439242 |  | 49 | -0.33 | -1.57 | 0.050 | 0.032 | 0.925 | 326 | tags=20%, list=8%, signal=22% |
| 88 | DOROSZKO\_U3180MG\_CLUSTER\_6 |  | 22 | -0.41 | -1.56 | 0.057 | 0.035 | 0.937 | 381 | tags=27%, list=9%, signal=30% |
| 89 | RICHARDS\_ZHONG\_NATURE\_2018\_MICROGLIA\_UPREG |  | 132 | -0.26 | -1.53 | 0.019 | 0.041 | 0.962 | 441 | tags=21%, list=11%, signal=23% |
| 90 | DOROSZKO\_U3054MG\_CLUSTER\_2 |  | 196 | -0.24 | -1.52 | 0.014 | 0.044 | 0.971 | 713 | tags=24%, list=17%, signal=28% |
| 91 | GANDAL\_ALCOHOLADDICTION\_UP\_PMID29439242 |  | 61 | -0.30 | -1.49 | 0.065 | 0.052 | 0.985 | 919 | tags=38%, list=22%, signal=48% |
| 92 | WANG\_CLASSICAL\_PMID\_28697342 |  | 16 | -0.41 | -1.43 | 0.090 | 0.071 | 0.998 | 1045 | tags=50%, list=25%, signal=67% |
| 93 | RICHARDS\_NOWAKOWSKI\_SCIENCE\_2017\_MURAL\_UPREG |  | 316 | -0.21 | -1.41 | 0.018 | 0.081 | 1.000 | 917 | tags=30%, list=22%, signal=36% |
| 94 | IVY\_CTPAN |  | 58 | -0.29 | -1.40 | 0.089 | 0.083 | 1.000 | 436 | tags=21%, list=11%, signal=23% |
| 95 | RICHARDS\_NOWAKOWSKI\_SCIENCE\_2017\_MICROGLIA\_UPREG |  | 323 | -0.20 | -1.38 | 0.018 | 0.096 | 1.000 | 618 | tags=20%, list=15%, signal=22% |
| 96 | RICHARDS\_LIDDELOW\_NATURE\_2017\_A2\_REACTIVE\_ASTROCYTES |  | 20 | -0.35 | -1.29 | 0.175 | 0.155 | 1.000 | 62 | tags=15%, list=2%, signal=15% |
| 97 | OLSEN\_MESENCHYMAL |  | 43 | -0.27 | -1.26 | 0.166 | 0.179 | 1.000 | 342 | tags=21%, list=8%, signal=23% |
| 98 | VANGRONINGEN\_MES |  | 227 | -0.19 | -1.24 | 0.111 | 0.190 | 1.000 | 725 | tags=22%, list=18%, signal=26% |
| 99 | NB\_KI\_CLUSTER6 |  | 162 | -0.20 | -1.20 | 0.203 | 0.236 | 1.000 | 1400 | tags=46%, list=34%, signal=67% |
| 100 | NB\_KI\_CLUSTER8 |  | 133 | -0.20 | -1.18 | 0.195 | 0.250 | 1.000 | 1641 | tags=55%, list=40%, signal=89% |
| 101 | RICHARDS\_NOWAKOWSKI\_SCIENCE\_2017\_GLYC\_UPREG |  | 312 | -0.17 | -1.17 | 0.169 | 0.270 | 1.000 | 1165 | tags=33%, list=28%, signal=43% |
| 102 | DOROSZKO\_U3054MG\_CLUSTER\_9 |  | 187 | -0.19 | -1.15 | 0.218 | 0.285 | 1.000 | 1539 | tags=49%, list=38%, signal=75% |
| 103 | ALMSTEDT\_11Q\_MSIGDB |  | 130 | -0.19 | -1.13 | 0.275 | 0.312 | 1.000 | 423 | tags=15%, list=10%, signal=16% |
| 104 | GANDAL\_BIPOLAR\_UP\_PMID29439242 |  | 45 | -0.24 | -1.11 | 0.287 | 0.334 | 1.000 | 262 | tags=16%, list=6%, signal=16% |
| 105 | GANDAL\_IBD\_UP\_PMID29439242 |  | 44 | -0.24 | -1.09 | 0.343 | 0.363 | 1.000 | 522 | tags=23%, list=13%, signal=26% |
| 106 | PARADA\_T1\_DN\_26461091 |  | 22 | -0.28 | -1.06 | 0.377 | 0.410 | 1.000 | 1095 | tags=45%, list=27%, signal=62% |
| 107 | PARADA\_T2\_DN\_26461091 |  | 20 | -0.29 | -1.06 | 0.389 | 0.406 | 1.000 | 1095 | tags=50%, list=27%, signal=68% |
| 108 | GANDAL\_IBD\_DN\_PMID29439242 |  | 18 | -0.29 | -1.05 | 0.369 | 0.415 | 1.000 | 1054 | tags=39%, list=26%, signal=52% |
| 109 | LARSSON\_STATE\_1 |  | 59 | -0.21 | -1.05 | 0.425 | 0.415 | 1.000 | 216 | tags=12%, list=5%, signal=12% |
| 110 | NB\_KI\_CLUSTER14 |  | 122 | -0.17 | -0.98 | 0.493 | 0.529 | 1.000 | 1464 | tags=46%, list=36%, signal=69% |
| 111 | RICHARDS\_VERHAAK\_CANCERCELL\_2010\_NEURAL |  | 28 | -0.22 | -0.92 | 0.568 | 0.638 | 1.000 | 1875 | tags=71%, list=46%, signal=131% |
| 112 | NB\_KI\_CLUSTER11 |  | 27 | -0.22 | -0.86 | 0.636 | 0.755 | 1.000 | 441 | tags=22%, list=11%, signal=25% |
| 113 | RICHARDS\_NOWAKOWSKI\_SCIENCE\_2017\_CHOROID\_UPREG |  | 248 | -0.13 | -0.82 | 0.816 | 0.809 | 1.000 | 465 | tags=12%, list=11%, signal=12% |
| 114 | GANDAL\_DEPRESSION\_DN\_PMID29439242 |  | 25 | -0.20 | -0.78 | 0.748 | 0.862 | 1.000 | 1008 | tags=36%, list=25%, signal=47% |
| 115 | NB\_KI\_CLUSTER13 |  | 72 | -0.13 | -0.70 | 0.885 | 0.938 | 1.000 | 592 | tags=17%, list=14%, signal=19% |
| 116 | RICHARDS\_NEFTEL\_CELL\_2019\_MES2 |  | 15 | -0.20 | -0.69 | 0.822 | 0.936 | 1.000 | 42 | tags=7%, list=1%, signal=7% |
| 117 | SUVA\_MES2\_PMID\_31327527 |  | 15 | -0.20 | -0.69 | 0.846 | 0.928 | 1.000 | 42 | tags=7%, list=1%, signal=7% |
| 118 | NB\_KI\_CLUSTER22 |  | 60 | -0.13 | -0.65 | 0.922 | 0.946 | 1.000 | 644 | tags=18%, list=16%, signal=21% |
| 119 | NB\_KI\_CLUSTER15 |  | 65 | -0.13 | -0.64 | 0.931 | 0.943 | 1.000 | 461 | tags=11%, list=11%, signal=12% |
Table: Gene sets enriched in phenotype **na **[plain text format]****
